# Skeletal Muscle Assessment Using Quantitative Ultrasound: A Narrative Review

**DOI:** 10.3390/s23104763

**Published:** 2023-05-15

**Authors:** Aria Ashir, Saeed Jerban, Victor Barrère, Yuanshan Wu, Sameer B. Shah, Michael P. Andre, Eric Y. Chang

**Affiliations:** 1 Department of Radiology, University of California, San Diego, CA 92093, USA; sjerban@health.ucsd.edu (S.J.); mandre@health.ucsd.edu (M.P.A.); ericchangmd@gmail.com (E.Y.C.); 2 Research Service, Veterans Affairs San Diego Healthcare System, San Diego, CA 92161, USA; vbarrere@health.ucsd.edu (V.B.); sbshah@health.ucsd.edu (S.B.S.); 3 Department of Radiology, Santa Barbara Cottage Hospital, Santa Barbara, CA 93105, USA; 4 Department of Orthopaedic Surgery, University of California, San Diego, CA 92093, USA; yuw100@ucsd.edu; 5 Department of Bioengineering, University of California, San Diego, CA 92093, USA

**Keywords:** skeletal muscle, ultrasound, quantitative ultrasound

## Abstract

Ultrasound (US) is an important imaging tool for skeletal muscle analysis. The advantages of US include point-of-care access, real-time imaging, cost-effectiveness, and absence of ionizing radiation. However, US can be highly dependent on the operator and/or US system, and a portion of the potentially useful information carried by raw sonographic data is discarded in image formation for routine qualitative US. Quantitative ultrasound (QUS) methods provide analysis of the raw or post-processed data, revealing additional information about normal tissue structure and disease status. There are four QUS categories that can be used on muscle and are important to review. First, quantitative data derived from B-mode images can help determine the macrostructural anatomy and microstructural morphology of muscle tissues. Second, US elastography can provide information about muscle elasticity or stiffness through strain elastography or shear wave elastography (SWE). Strain elastography measures the induced tissue strain caused either by internal or external compression by tracking tissue displacement with detectable speckle in B-mode images of the examined tissue. SWE measures the speed of induced shear waves traveling through the tissue to estimate the tissue elasticity. These shear waves may be produced using external mechanical vibrations or internal “push pulse” ultrasound stimuli. Third, raw radiofrequency signal analyses provide estimates of fundamental tissue parameters, such as the speed of sound, attenuation coefficient, and backscatter coefficient, which correspond to information about muscle tissue microstructure and composition. Lastly, envelope statistical analyses apply various probability distributions to estimate the number density of scatterers and quantify coherent to incoherent signals, thus providing information about microstructural properties of muscle tissue. This review will examine these QUS techniques, published results on QUS evaluation of skeletal muscles, and the strengths and limitations of QUS in skeletal muscle analysis.

## 1. Introduction

Ultrasound (US) has been an invaluable medical imaging tool for decades. Its accessibility, absence of ionizing radiation, dynamic imaging, cost-effectiveness, and various imaging modes are important strengths.

The dominant ultrasound technique uses reflection or echo-ultrasonography where a transducer emits ultrasound waves and captures returning signals from macro- and micro-structures within and between tissues. The sound waves are created by transducers, which are stimulated by electric pulses to vibrate at desired frequencies, usually ranging between 2 and 18 MHz in most clinical settings. The transducers typically use piezoelectric ceramics or crystals, but some recent systems use silicon microchips containing, for example, capacitive micromachined ultrasound transducers (CMUTs). The phased-array beamforming technique can be used where individual or groups of transducer elements are energized with specific delayed transmit–receive sequences, allowing for focusing of the sound waves at a desired depth and steering of the beam [1]. 

**Figure 1:**
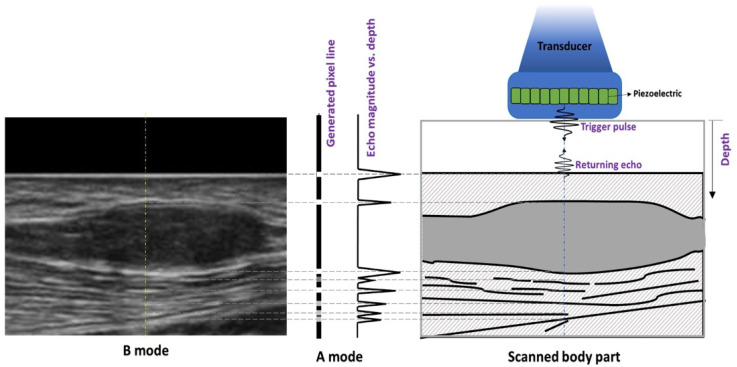
Graphic illustrating A- and B-mode data generation in ultrasonography. A piezoelectric element within an ultrasound probe resonates, generating an acoustic pulse. Interfaces and scatters in the tissue are encountered, and the returning echoes may be displayed as A-mode data, a pixel line, or a B-mode image.

Clinical ultrasound utilizes multiple imaging modes including A-mode, B-mode, M-mode, and Doppler-mode [1]. In amplitude modulation mode (A-mode), a transducer sends an ultrasound pulse along a line through the tissue, after which echoes are received back to the transducer from interfaces that the sound wave encounters. The echoes are then plotted as echo intensity amplitude vs. time. Time is converted to depth in the tissue on the line of propagation based on the round-trip time for an echo and assuming a constant speed of sound in the tissue, usually 1540 m/s for muscle. A-mode imaging has been used to measure muscle thickness [2,3], but compared with the other modes, it is seldomly used in the musculoskeletal ultrasound clinic. In brightness mode imaging (B-mode), a transducer scans a plane to produce a two-dimensional (2D) sonographic image that is reconstructed from multiple lines of A-mode amplitude vs. depth data. The amplitudes obtained from the A-mode data are coded in shades of gray to ease interpretation, where the higher and lower amplitudes appear as brighter and darker pixels, respectively. The tissue contrast (range of echo amplitudes) is dependent on the differences in tissue acoustic properties including the density, sound speed, relative transmission, scattering, and absorption encountered by propagated sound waves [4]. B-mode imaging is the most utilized in the clinical setting, including for analysis of muscles. Figure 1 presents the concepts of A-mode and B-mode imaging in a schematic fashion.

Motion modulation (M-mode) is based on displaying the echoes from a moving structure by plotting the A-mode information as a function of time. M-mode recordings conventionally display time on the x-axis and distance from the transducer on the y-axis, measuring a line of interest continuously. M-mode imaging has been used to detect skeletal muscle activity and thickness changes during muscle contraction [5,6]. However, M-mode imaging is more commonly used for assessment of cardiac muscle for evaluation of heart chambers and valves (echocardiography). Lastly, Doppler-mode imaging utilizes the Doppler effect, which is the change of a sound wave frequency due to a reflector moving towards or away from the transducer. Structures moving towards the transducer will return echoes shifted higher than the transmitted frequency, and structures moving away from the transducer will return echoes with decreased frequency. The degree of shift is used to compute the velocity of the moving structures. Velocity can be mapped onto the B-mode images to visualize both the anatomy and motion. Color-coded maps use shades of blue to indicate speed of motion away from the transducer (negative) and shades of red for motion towards the transducer (positive), thereby depicting both flow speed and direction. This is helpful for characterizing vasculature, including that within muscles [7]. Doppler information is quantitative and useful for analyzing velocity waveforms, which provide information about vessel flow, turbulence, and potential stenosis.

Ultrasound supports several clinical applications for evaluation of skeletal muscles. Many diseases, including neuromuscular pathologies and injury, can cause muscle changes in the form of fatty infiltration and fibrosis [8,9]. Ultrasound can evaluate muscle composition, physiologic parameters (e.g., pennation angles), post-traumatic muscle integrity, and muscle edema [10]. Compared with other imaging techniques, real-time ultrasound also allows for dynamic evaluation of muscle fasciculations and contractions [9]. However, most such evaluations are qualitative, and are limited due to a lack of objective measures and both scanner- and operator-dependent bias. Although quantitative ultrasound (QUS) is not completely free of these limitations, the ability to obtain more objective and repeatable measures, as well as utilize raw backscattered ultrasound data, has created potential advantages for these techniques. This review focuses on four main categories of QUS, which include measurements made on B-mode images, ultrasound strain and shear wave elastography, radiofrequency (RF) signal characterization, and envelope statistics-based methods. We survey normal skeletal muscle anatomy on ultrasound images, the relevant medical physics associated with each technique, the quantitative muscle ultrasonography literature, and the limitations associated with quantitative ultrasound methods.

## 2. Normal Skeletal Muscle Anatomy on Ultrasound

There are over 200 muscles in the human body with varying morphology and architecture. Muscles are formed by individual fibers called myofibers, within which lie contractile elements (sarcomeres). Myofibers are surrounded by the perimysium into bundles (fascicles). The fascicles comprise the muscle “belly,” which is itself surrounded by the dense fibrous tissue known as the epimysium. Individual muscle fibers can have a wide range of length, from a few millimeters in the stapedius to 50 cm in the sartorius [11]. Muscles can also have various shapes including fusiform, triangular, strap or digastric, which are dictated by the organization of muscle fibers. Fibers may run directly from origin to insertion of the muscle, or obliquely (pennate), with the number of fiber orientations determining whether muscles are unipennate, bipennate, or multipennate. Muscles can attach directly to the periosteum of the bone or be attached by tendons, with the musculotendinous junction representing a specialized interface between muscle and tendon. This unit plays an important role in how force is transmitted between two tissues and is a common area for strain injuries in sports [12].

**Figure 2:**
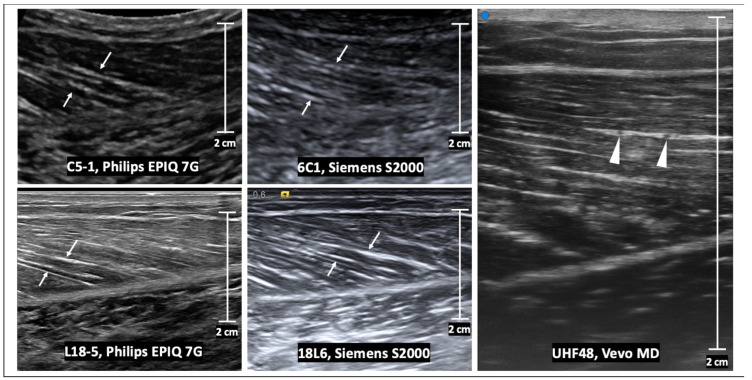
Longitudinal B-mode ultrasound images of a medial gastrocnemius muscle in a healthy volunteer. The echogenic perimysia are evident on all images (arrows), but the appearance differs with varying transducer frequency and vendor reconstruction algorithms. At ultra-high frequencies (e.g., using the UHF48 transducer), intramuscular veins are evident within a layer of the perimysium (arrowheads). Scale bars represent 2 cm.

**Figure 3:**
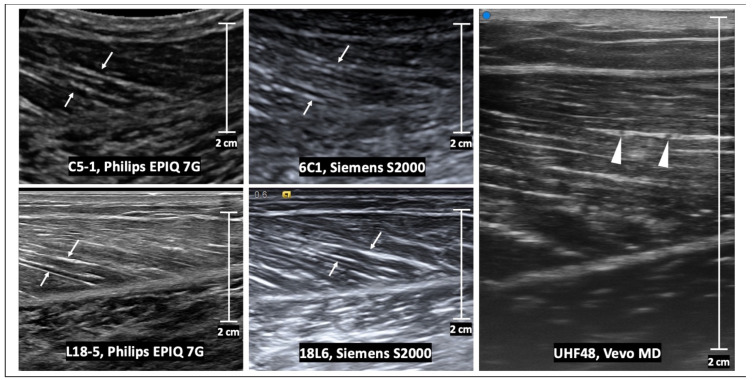
Transverse B-mode ultrasound images of the same normal muscle as in Figure 2. The internal muscle architecture is normal in all images, but the appearance differs with the transducer frequency as well as the vendor reconstruction algorithm. At low frequencies (e.g., using C5-1 and 6C1 transducers), there are subtle echogenic bands which represent the perimysium (arrows). At high frequencies (e.g., using L18-5 and 18L6 transducers), the echogenic perimysium is more apparent and the interfaces are better delineated. At ultra-high frequencies (e.g., using the UHF48 transducer), there is further improved resolution of the arborizing pattern of perimysium. Scale bars represent 2 cm.

The appearance of normal muscle on ultrasound consists of hypo-reflective muscle fascicles that have hyper-reflective connective tissue separating them. In a longitudinal plane, perimysial borders are seen as linear bands within the muscle belly, and in the transverse plan these are seen as dots and bands [13] (Figure 2 and Figure 3). The epimysium and tendon structure display increased reflectivity. Tendons are fibrillar structures appearing as multiple echogenic stripes. This echogenicity can appear reduced if the ultrasound probe is not parallel to the tendon, due to reflection angle and anisotropy, the tendency of an object to be directionally dependent. Anisotropy also affects endomysial and perimysial fibers, and should be taken into consideration during muscle ultrasound analysis [13]. High-frequency probes are needed (usually greater than 7 MHz) to adequately image muscle structure, as increasing frequency increases axial spatial resolution. Compared with curved array probes, linear probes reduce distortion due to sound divergence with increasing tissue depth and improve the lateral resolution, but with smaller fields of view [13].

## 3. B-Mode Qualitative and Quantitative Techniques

B-mode images depict both tissue reflections and scattering. Reflections occur at tissue interfaces, which are defined by discontinuities in tissue density and/or sound speed. When the tissue interface is large compared with the beam wavelength and smooth, the reflection is referred to as specular and occurs with the interface displayed as hyperechoic. B-mode images also contain information in the form of backscattered signals from the microstructure of the tissues smaller than the wavelength of the transmitted sound waves. The backscattered energy reemitted by the small structures appears as speckle texture in the images, arising largely from diffraction. Relatively brighter structures on B-mode from these combined processes are referred to as echogenic or hyperechoic, and darker structures as hypoechoic.

Qualitative B-mode ultrasound imaging is the mainstay of clinical evaluation; however, there are several challenges to subjectively analyzing muscles. The ultrasound signal can be highly dependent on the system settings such as gain, location of focal points, and vendor-dependent post-processing algorithms. Muscle tissue properties and echogenicity may change based on factors including weight, age, hand-dominance in the upper extremity muscles, and nutritional status. In aging adults, there are changes due to the increased fibrotic content of muscles, known as sarcopenia [14,15]. Obesity also leads to increased subcutaneous and intramuscular fat, which displays increased echogenicity and higher attenuation of acoustic signal due to overlying tissues above the target muscle [16].

Qualitative analysis of these muscle changes can be difficult, but there have been efforts to create more objective methods for evaluating echogenicity. For example, the Heckmatt scale utilizes a four-point visual grading scale based on the relative muscle gray-scale appearance compared with that of the overlying subcutaneous fat layer, the presence or absence of a specific muscle architecture, and the degree of attenuation leading to less underlying bone or fascia echogenicity [8]. There remains a significant component of subjectivity associated with this technique; its reported sensitivity is 71–76% [17,18]. Pillen et al. compared the sensitivity and specificity of visual evaluation and quantitative gray-scale echo intensity measurements of skeletal muscle in children suspected of having neuromuscular disorders using a strong reference standard (medical history and clinical investigation, electromyography, biochemical evaluation, genetic evaluation, and muscle biopsy). The Heckmatt criteria were utilized for visualization, and the gray-scale analysis of muscle echo intensity was obtained in the biceps brachii, forearm flexors, quadriceps femoris, and anterior tibial muscles. Visual evaluation was found to have a sensitivity of 71% and specificity of 92%. Meanwhile, gray-scale analysis showed a sensitivity of 87% and specificity of 67% and higher inter-observer agreement than the visual method. Given the higher sensitivity and higher inter-observer agreement, gray-scale analysis may provide an effective screening tool for diagnosis of neuromuscular disease in children [19]. 

The analysis of B-mode images using quantitative methods can offer more objective techniques to assess muscles. B-mode echogenicity measurements utilize techniques to examine the microstructure of tissue. A common method involves obtaining the mean gray-scale level within a manually selected region of interest (ROI) of an image and comparing it to reference values obtained from a specific muscle. The measured gray-scale values can be transformed into Z-scores, which represent the standard deviations from the reference values for the mean echogenicity of that muscle (Z-score of 0 being identical to the mean echogenicity value, Z-score of 1 being one standard deviation from the mean echogenicity value, Z-score of 2 being two standard deviations from the mean echogenicity value) [19]. The mean gray-scale values can be calculated from pixel histogram analysis (e.g., ImageJ https://imagej.nih.gov/ij/ (accessed on 19 March 2023)) [20] or Photoshop (Adobe Systems, San Jose, CA, USA) [8,21]. Different ROI acquisition tools such as the rectangular marquee tool (RMT) and the free hand tool (FHT) have shown high reliability with both ImageJ and Photoshop [21]. Another QUS method reliant on B-mode images is spatial frequency analysis (SFA). This method evaluates the anisotropic two-dimensional B-mode speckle pattern of a tissue region in the spatial frequency domain. The spectral parameters are related to information about the underlying tissue organization [22]. Ultrasound B-mode images can also be used to estimate the pennation angle, muscle thickness and length, and fascicle angle to characterize anatomy and architecture [23,24].

Pathological conditions affecting muscles may lead to interstitial fibrosis and fatty atrophy. Echo intensity measurement has been shown to be a reproducible technique for measuring intramuscular fat [25]. Reimers et al. examined whether high echo intensities are caused mainly by interstitial fat or fibrosis using echo intensity measurements, morphometry, and biochemical testing. Fatty replacement was found to correlate better with increased muscle echogenicity than fibrosis [26]. Interestingly, Pillen et al. compared quantitative echo intensity values with muscle structure in golden retriever dogs with muscular dystrophy and found a significant correlation between echo intensity and interstitial fibrosis [27]. Similarly, mean echo intensity in injured fibrotic gastrocnemius muscles of rats was higher than the unaffected muscles [28].

Various muscles have been evaluated with B-mode echogenicity measurements. Arts et al. investigated echo intensity values in several normal muscles and found correlations with age, gender differences, and laterality differences in the upper extremities [15]. Gao et al. utilized both B-mode echogenicity techniques and strain ultrasonography to assess the biceps brachii and gastrocnemius muscles in multiple sclerosis (MS) patients. Mean muscle pixel intensity in gray-scale images of affected muscles in MS patients was compared with unaffected muscles in MS patients and muscles in healthy volunteers. Significant differences were found between these groups, suggesting mean echo intensity may be used for assessing muscle echogenicity in adults with multiple sclerosis [29]. Wilkinson et al. used gray-level co-occurrence matrix (GLCM), a mathematical toolset used for image texture analysis, to demonstrate that differences in muscle homogeneity correlated with muscle quality in patients with chronic kidney disease, with increased image homogeneity corresponding to less fat infiltration and fibrosis [30]. Nielsan et al. used both mean intensity measurements and texture analysis of ultrasound images to study the supraspinatus and vastus lateralis muscles. They found higher gray-scale intensity and homogenous regions in the vastus lateralis muscle, indicating that the thigh contains more non-contractile components and was coarser than the supraspinatus. A more complete description of tissue composition was obtained than when using gray-scale values alone [31]. Oranchuk et al. demonstrated varying echo intensity values between the proximal and distal regions of the quadriceps, with the mid-region being the lowest, likely because of an increase in fibrous content towards the myotendinous junction and tendon ends. Furthermore, the authors found that subcutaneous fat correction substantially decreased variability [32]. 

Kitaoji et al. assessed the utility of automatic thresholding methods for muscle echogenicity measurements in patients with Charcot–Marie–Tooth disease type 1A (CMT1A). The abductor pollicis brevis, first dorsal interosseous, biceps brachii, tibialis anterior, gastrocnemius, and vastus lateralis were assessed using both conventional mean gray-scale analysis and an automated thresholding method. In the automatic thresholding algorithms, all the pixels were segmented into black or white based on the threshold calculated by the gray-scale histogram of the original image, and the proportion (%) of the white area (hyperechoic fraction) in the ROI was calculated. Significant differences were found in the mean echogenicity values of these muscles when comparing CMT1A patients with controls utilizing gray-scale image analysis and in eleven of sixteen automated thresholding methods. Interestingly, eight out of the sixteen automated thresholding methods correlated with the Charcot–Marie–Tooth neuropathy score, while none of the results did for the gray-scale image analysis. Thus, automated thresholding tools may detect differences between CMT1A patients vs. controls and provide values which correlate with disease severity [33]. Additional studies have found that echo intensity measures of the first dorsal interosseous and tibialis anterior are useful disease markers in CMTA1A patients [34]. Automatic thresholding methods have also been found to supplement qualitative image analysis in amyotrophic lateral sclerosis [35].

There has been research on the use of SFA QUS methods to obtain information from B-mode images. SFA has previously been used to evaluate tendons [36,37,38,39,40,41]. This technique has been applied to muscles given their similar anisotropy to tendons due to parallel striations of hypoechoic fibers and the hyperechoic perimysium. Crawford et al. investigated the reliability of spatial frequency parameter measurements for a muscle group in healthy subjects. The inter-class correlation coefficients showed high intra- and inter-rater reliability and good to moderate test-retest reliability [42]. An additional study showed SFA could detect architectural differences in multiple hamstring muscles with significant differences between different regions of healthy muscle structure [22]. A follow-up study used SFA to analyze injured hamstrings and provided a quantitative assessment of fascicular disruption and the presence of edema in hamstring injury. Some parameters were found to show significant differences when compared with uninjured regions while others showed no none [43].

Echo intensity values have been combined with measurements of the muscle thickness to track changes that occur during a strength training program [44]. A long head of the biceps femoris fascicle with short length and low eccentric knee flexor strength was found to increase the risk of hamstring strain injury in professional soccer players [45]. Ultrasound has been shown to be a reliable and valid tool for assessing muscle cross-sectional area [46]. The semitendinosus and biceps femoris long head have been shown to have varying architecture along their length [47]. A combination of echo intensity measurements, muscle–tendon length, tendon cross-sectional area, volume, and length measurements for hamstring muscles have elucidated information about each individual muscle and its architecture, which may have implications for exercise, injury, and recovery [48,49].

Various protocols for B-mode QUS scanning have been studied. Nielsen et al. validated a quantitative image analysis protocol for analyzing images of the supraspinatus muscle. Gray-scale statistics were used to evaluate muscle composition in fourteen healthy patients. Data from one scanning site were not representative for the whole muscle due to inhomogeneity. A scanning session consisting of three different scanning sites along the muscle in both transverse and longitudinal directions was found to provide higher day-to-day reproducibility [50]. Obst et al. found that increased muscle thickness in young, typically developing children was related to regional variations in muscle echogenicity. The normalization of muscle echogenicity to muscle thickness may provide a useful measure of muscle echogenicity when comparing muscles of different cross-sectional areas [51].

Echo intensity measurements have also been compared using magnetic resonance imaging (MRI) techniques in evaluating fatty infiltration of the supraspinatus muscle in patients with rotator cuff tears. MRI was used to identify the extent of tear size and fatty infiltration using the Goutallier classification. The muscles were then assessed for their echo intensity. The supraspinatus echo intensity was significantly lower for stage 0 and stage 1 Goutallier tendons than for those with higher degrees of fatty infiltration and tearing. These results suggest that ultrasound can assess fatty infiltration in rotator cuff muscles [52].

## 4. Ultrasound Elastography

Ultrasound elastography was originally described by Ophir et al. in 1991 [53]. Since then, elastography has been used in various applications, for example, in the evaluation of liver fibrosis [54]. Elastography is analogous to palpation used during a physical exam. Palpation involves the examiner manually tapping/shearing the tissue and qualitatively feeling for stiffness or deformability. Similarly, ultrasound elastography analyzes the deformation of tissue in response to an applied force. The two major techniques for ultrasound elastography are strain and shear wave elastography (SWE). Strain ultrasonography utilizes compression with detection and tracking of speckle displacement on B-mode images [55,56]. SWE involves measurements of the speed of induced shear waves produced by external or endogenous sources, which yields estimations of tissue elasticity [55,56].

**Figure 4:**
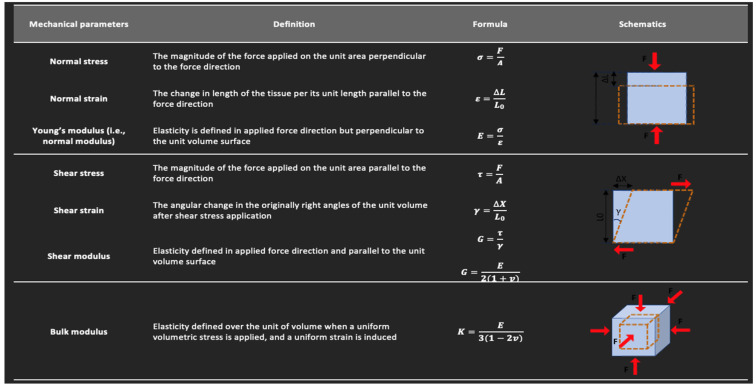
Definitions of the basic mechanical parameters described in this study. This figure is adapted from a figure presented by Jerban et al. [60] and reprinting is permitted under the Creative Commons CC BY license.

Strain and elasticity are important concepts to review in US strain imaging and SWE. Strain is described as the change in length of a tissue, relative to its original length, that results from an applied stress [57]. Stress is defined as the magnitude of the applied force divided by the tissue’s cross-sectional area. Elasticity can be described as the tissue’s resistance to shape and volume changes under an applied force [57]. The ability of solids to withstand changes in shape and volume is due to their possession of shear and volume elasticity. In contrast, liquids possess volume elasticity, but no shear elasticity since their shape changes under an applied shear stress (i.e., shear stress results in a velocity gradient in liquids depending on their viscosity) [58]. Biological tissues are usually considered as viscoelastic material as they also possess both an elastic component (often modeled as a spring) as well as a time-dependent viscous element (often modeled as a dashpot) in their response. Therefore, they contain both shear and volume elasticities, though the volume elasticity is much greater than the shear elasticity [59]. The elasticity modulus can be quantified based on the stress and strain direction. Young’s modulus, (E), equal to stress divided by strain, quantifies a material’s stiffness and its ability to resist deformation in the perpendicular direction when subjected to an external force. Shear modulus, G, represents the ability of a material to resist shear deformation, which arises when two parallel forces are applied to opposite surfaces of a material [57]. The basic mechanical properties discussed in this paper and their definitions are summarized in Figure 4.

### 4.1. Strain Elastography

**Figure 5:**
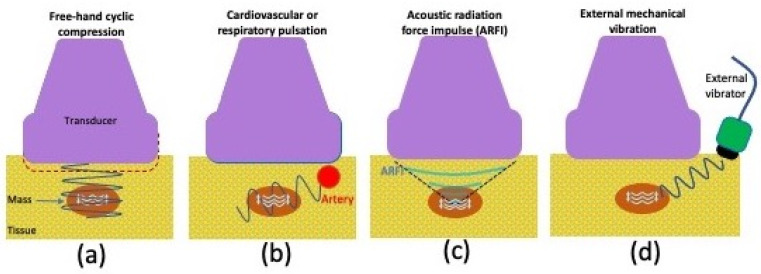
Graphic illustrating various strain ultrasonography techniques, including (**a**) using cyclic compression with a free hand, (**b**) pulsation from internal organs such as the heart or lungs, (**c**) acoustic radiation force impulse, or (**d**) vibration from an external source.

Strain elastography assesses the induced tissue displacement in the direction of the applied stress. There are four methods (Figure 5) for applying the required cyclic compression: (a) free-hand cyclic compression (palpation); (b) passive elastography using shear waves naturally present in the body, such as those resulting from cardiovascular pulsation or respiratory motion; (c) acoustic radiation force impulse (ARFI); or (d) external mechanical vibration [61]. The induced strain can then be measured with various methods including radiofrequency (RF) echo correlation-based tracking, Doppler processing, or a combination of these [61]. In RF echo correlation-based tracking, the RF lines are acquired along the axis of displacement, and their changes between different acquisitions are used to measure tissue displacement and thereby assess the elasticity of a tissue.

The free-hand cyclic compression technique involves the operator compressing and releasing force with the ultrasound transducer. The frequency and amount of force are adjusted by the operator who follows a target range marked by an indicator on the scanner screen. It can be difficult to control applied stresses when using free-hand compression or internal body pulsation techniques; thus, strain ratios (SR) are frequently calculated to produce more reliable indices.

ARFI-based compression utilizes a high intensity (e.g., spatial peak pulse average equal to 1400 W/cm^2^, spatial peak-temporal average equal to 0.7 W/cm^2^) and a long duration (i.e., 0.1–0.5 ms vs. 0.02 ms pulses in B-mode imaging) pushing pulse to displace tissue about 10–20 μm along the acoustic axis. Then, the magnitude of applied acoustic radiation force per unit of surface, *F*, is calculated according to Equation (1) [55,56,61]:

(1) $$F=\frac{2\alpha I}{c},$$

where *α* is the acoustic absorption rate in the tissue, *c* is the sound speed within the tissue, and *I* is the acoustic beam temporal average intensity.

### 4.2. Strain Elastography Applied to Muscles

There has been notable research on muscle evaluation using various ultrasound strain elastography techniques. Shimoyama et al. assessed the reliability of ultrasound strain elastography with an acoustic coupler for evaluating stiffness of the trapezius and supraspinatus muscles in young healthy volunteers. They found substantial reliability, but also identified a need for devices that can standardize scanning techniques for even greater reliability [62]. Oppersma et al. conducted a study using ultrasound speckle tracking to evaluate the diaphragm during inspiratory loading. They found this technique to be a potentially useful method for tracking diaphragm effort, which could have important indications in intensive care unit patients being evaluated for extubation [63]. Aşkın et al. used a free-hand strain elastography technique to study biceps brachii muscle stiffness in stroke patients and the correlation with clinical parameters following botulinum toxin-A (BTA) injections. They found higher strain indices (biceps muscle compared with subcutaneous fat tissue) on the affected side compared with the unaffected side. There were statistically significant differences in the modified Ashworth scale (a subjective tool for measuring spasticity) and strain indices between the pre- and post-treatment periods. However, there was no statistically significant correlation between clinical parameters (duration of disease, number of injections, goniometric injections) and strain indices. These results support the potential use of strain elastography as a diagnostic tool for assessing stiffness in spastic muscles and may help with establishing treatment plans and monitoring therapeutic responses to BTA injections [64].

Gao et al. investigated the feasibility of strain elastrography for the assessment of resting muscle stiffness in Parkinson’s disease patients. They used an external compression technique using a 1.5kg sandbag and 2D speckle tracking to estimate deformation of the biceps brachii muscles and the subcutaneous tissues as a reference in order to obtain a strain ratio. Significant differences in strain ratio were observed between the Parkinson’s disease and healthy control groups. There was a negative correlation between the strain ratio and the unified Parkinson’s disease rating scale. These results suggest that the strain ratio can be used to evaluate resting muscle stiffness in Parkinson’s disease patients suffering from muscle rigidity [65]. In a similar study, muscle stiffness was evaluated using strain elastography in patients receiving an acute levodopa challenge, which is given for confirmation of a potential Parkinson’s disease diagnosis. There was an increase in strain ratio in patients receiving the levodopa challenge, suggesting that strain elastography may be used to evaluate the effect of levadopa on muscle stiffness in the diagnosis of Parkinson’s disease [66].

### 4.3. Shear Wave Elastography

Shear wave elastography (SWE) uses ultrasound imaging to produce mechanical waves that travel perpendicular to the direction of the ultrasound beam. The shear wave speed (SWS) is then measured to estimate the shear elastic modulus (*G*) and normal elastic modulus (*E*) [56], as presented in Equation (2):

(2) $$SWS=\sqrt{\frac{G}{\rho }}\approx \sqrt{\frac{E}{3\rho }},$$

where *ρ* represents tissue density.

Therefore, SWS can be used to estimate the elastic modulus of a tissue. Soft tissues have an average SWS < 10 m/s. Shear waves travel faster if the tissue is stiffer, slower if it is denser, and faster along the tissue structural axis, such as along fascicles of tendons [67].

**Figure 6:**
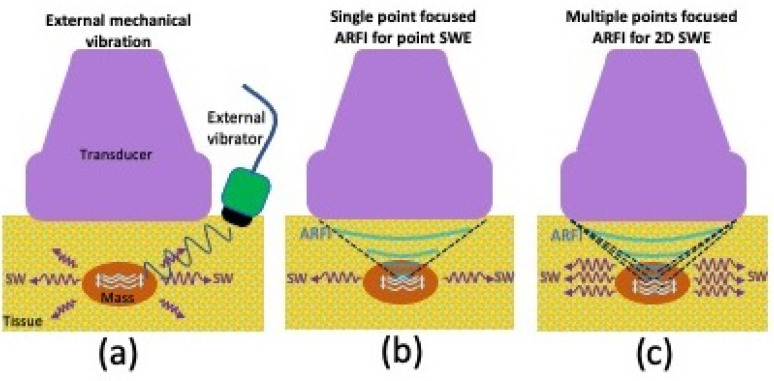
Graphic illustrating various shear wave elastography (SWE) techniques, including (**a**) vibration from an external mechanism, (**b**) single-point focused acoustic radiation force impulse as with point-SWE, or (**c**) multiple-point focused acoustic radiation force impulse as with 2D-SWE.

The methods for generating shear waves include vibration from an external mechanism, as well as ARFI that is focused at a single or multiple points [61]. These methods are schematically presented in Figure 6. The external mechanical vibration was first proposed by Krouskop et al.; however, most clinical studies utilize shear waves induced by ARFI pulses as initially proposed by Sugimoto et al. [68,69]. An ARFI pulse perpendicular to the tissue surface results in tissue vibration at an ultrasonic frequency and consequent tissue deformation (displacement) within the region of ultrasound excitation, due to sound wave absorption and scattering. The resultant tissue displacement converts some of the energy to shear waves that travel laterally away from the region of excitation at a much lower velocity (<10 m/s) when compared with ultrasound pressure pulses (1500 m/s). Although there is shear wave generation during B-mode imaging, the force magnitudes are too small to create tissue motion detectable by conventional US. ARFI pulses can be applied to a single focal location (point shear wave elastography). They can also be applied to multiple points such that each focal zone is interrogated in rapid succession, creating a cylindrical-shaped shear wave extending over a large depth and allowing for real-time shear wave images to be formed. The latter technique is also known as 2D shear wave elastography (2D-SWE) [55,56,61]. 2D-SWE uses shorter propagation distances due to limitations in the number of tracking pulses.

If the generation of shear waves is considered the first step of ARFI SWE techniques, the second step uses US fast plane wave excitations to track tissue displacement due to the propagation of shear waves. The third step consists of using changes in tissue displacement maps over time to calculate the SWS, with tracking frame rates typically between 2 and 10 kHz [67]. Scanners display the quality index as a measure of confidence in the SWS. This is calculated from the correlation coefficients between frames of the speckle tracking images. A lower frame rate, patient or probe motion during the US exam, and a lack of well-developed speckle are factors that can contribute to decreased correlation of speckles between frames, and thus, a decreased quality index.

### 4.4. Shear Wave Elastography Applied to Muscles

Several studies have focused on measuring muscle elasticity in healthy individuals. Le Sant et al. assessed the individual behavior of hamstring muscles (semitendinosus, semimembranosus, and the long and short head of the biceps femoris) in different stretching positions using SWE. They found increased shear modulus with increased stretching in the semitendinosus, semimembranosus, and the long head of the biceps femoris. The short head of the biceps femoris did not show the same results, suggesting the importance of probe placement relative to the key elements of muscle architecture; in this case, differences were postulated to be due to the transducer placement near the stiff tendon of the nearby long head and due to the muscle–tendon unit being monoarticular. Interestingly, the shear modulus values were found to be different for each muscle for the same amount of perceived stretching at different degrees of hip flexion. The highest shear modulus was in the high flexed-hip position, signifying that hip flexion levels may be fundamentally tied to efficient stretching in the muscle–tendon component during passive knee extension [70]. Another study found SWE to be a reliable tool for assessing hamstring stiffness during isometric muscle contraction [71]. Brandenburg et al. investigated the use of SWE in assessing passive muscle stiffness in healthy children. They analyzed the bilateral lateral gastrocnemius muscles in four positions of progressive passive foot dorsiflexion. They found increased stiffness with increasing stretch. There was no significant difference when comparing laterality of the extremities or between genders [72]. Eby et al. quantified the shear modulus of the biceps brachii in different positions in adults of various ages. The shear modulus values were found to increase with age. Interestingly, the shear modulus values were higher for females than males, even though there are established trends of males having higher passive joint torque. This suggests that joint torque may be related to other factors in addition to the shear modulus/stiffness of muscles [73]. Hirata et al. used SWE to study the stiffness responses of the medial and lateral gastrocnemii and soleus before and after passive dorsiflexion. Interestingly, this study found the stiffness of the medial gastrocnemius to be higher than the soleus muscle, although previous non-ultrasound measures of stiffness have shown the soleus to be stiffer. This may be due to the ability of SWE to measure isolated stiffness of the muscle, while other methods may be affected by stiffness from the surrounding connective tissue structures [74].

There is notable literature on the use of SWE in assessment of various pathological conditions. Lacourpaille et al. used SWE to evaluate stiffness in relaxed muscles of patients with Duchenne muscular dystrophy (DMD). Stiffness was found to be higher in DMD patients compared with controls. The increased stiffness was found to be similar in both the stretched and shortened positions in multiple muscles, which is significant for evaluating patients with high degrees of joint contractures who cannot otherwise be scanned in a stretched position. These findings suggest that SWE may be a non-invasive tool for evaluating disease progression and treatment results, and adapting physiotherapy treatments for specific muscles [75]. Ding et al. used SWE to assess muscle rigidity in Parkinson’s disease patients. They found the mean shear wave velocity (SWV) of the biceps brachii and brachioradialis muscles were higher in the Parkinson’s disease group than the control group. SWV was found to be associated with joint rigidity and disease duration, and thus, may serve as an objective quantitative tool for evaluating rigidity [76]. Burke et al. studied SWV changes within the pronator quadratus muscle in patients following volar plate fixation of distal radius fractures compared with the contralateral nontreated side. There was a significant reduction in the SWV of the treated side compared with the nontreated side. This was thought to be due to fatty infiltration and atrophy leading to less stiffness and a decreased SWV. SWV values may also be useful in the assessment of postop changes in muscle, and this technique may provide the ability to compare with internal controls for rehabilitation monitoring [77]. Studies have found SWE helpful in evaluating fatty degeneration of muscle and found it to correlate well with results obtained by MRI and conventional ultrasound [78]. A few studies have assessed the use of SWE in rotator cuff muscle evaluation. Itoigawa et al. used SWE combined with B-mode imaging to measure in vivo stiffness of the supraspinatus [79]. Hatta et al. used SWE to assess the extensibility of the supraspinatus musculotendinous unit in cadaveric specimens including intact and torn groups. There was a negative correlation between the SWE modulus and extensibility measured experimentally; thus, SWE may help in predicting the extensibility of rotator cuff tears for pre-surgical planning [80]. The SWV of the supraspinatus muscle has been shown to decrease with increased fatty infiltration of the muscle, with elastography showing high reliability in assessing tendon and muscle quality [81]. SWE and echo-intensity measurements have both been used to assess muscle properties in polymyositis (PM) and dermatomyositis (DM) patients. PM and DM patients had increased muscle echo-intensity compared with controls. The SWV in the longitudinal orientation in PM and DM patients was significantly decreased, except for the biceps brachii muscle. Both echo-intensity measurements and SWE may serve as biomarkers in the diagnosis of PM and DM where there was found to be decreased muscle thickness, increased echo-intensity, and decreased SWV values in diseased patients [55].

### 4.5. Considerations for Evaluating Muscles with Shear Wave Elastography

It is important that studies are carried out to evaluate the normal baseline measurements of muscles. Even in a specific anatomical muscle group, each individual muscle can have significantly different baseline stiffness values. The trapezius, levator scapulae, anterior scalene, and sternocleidomastoid quantitative values were found to differ significantly, with the trapezius having higher values even in healthy volunteers [82].

Studies suggest that scanning orientation can also affect SWE measurements. The transverse scanning orientation has been shown to provide higher SWV than the longitudinal orientation, even for the same muscles [83]. The SWV has also been shown to increase with increasing depth [84]. These findings suggest that scanning should be performed at approximately the same depth throughout an examination. Studies also show that patient positioning can affect SWV measurements, likely due to postural influences on muscle/fascicle length [82,85]. Different ultrasound acquisition methods have been studied, including variation in ROI sizes and orientations (i.e., transverse, longitudinal, and oblique). A medium-sized 75 mm^2^ ROI and longitudinal orientation were found to produce higher internal agreement. [86]. Altering SWE acquisition methods can produce variable results; therefore, further research into these topics is warranted.

## 5. Radiofrequency Spectral-Based Characterization

Some ultrasound scanners provide a research interface that permits recording of the beam-formed RF signal prior to image formation. The quantitative characterization of RF spectral data enables measurement of fundamental acoustic tissue parameters from the normalized power spectrum, including the attenuation coefficient (AC), backscatter coefficient (BSC), and stochastic modeling of backscattered echoes in the far field [87,88]. These parameters are dependent on the composition and microstructure of the tissue, and the frequency of the ultrasound, and may be scanner independent [87,88]. Another fundamental tissue acoustic property is the speed of sound. Its measurement is applied clinically for assessing bone stiffness or mineralization [89,90]. However, in muscle, robust estimation of sound speed requires the ultrasonic array to be driven by a fully programmable electronic system, and most clinical scanners are not yet able to support this method.

The use of RF signals has several potential advantages. Not only do RF signals contain more information than B-mode images or the envelope data [4], they are also less dependent on system settings and post-processing operations or can be corrected for these, which can reduce variability [4]. For instance, RF signals are not influenced by the dynamic range setting and filtering operations that affect the appearance of B-mode images. Additionally, diagnostic techniques based on RF signals are potentially more suitable for devices that do not easily produce B-mode images, e.g., emerging wearable ultrasound devices [91,92].

BSC with units of 1/cm-sr represents the energy returned to the transducer from the tissue as a fraction of emitted signal and is analogous to echogenicity assessed qualitatively. AC (dB/cm-MHz) is a measure of ultrasound energy loss due to absorption, scattering and reflection in tissue and is observed in B-mode images as a loss of contrast, obscuration or blurring of structures deep in tissue. To assess the BSC, the power spectrum must be compensated for attenuation. The diffraction of the transducer, receiver gain and processing settings of the scanner are additional factors. The effect of beam diffraction and machine settings can be accounted for by utilizing the reference phantom technique, in which measured values are normalized to a calibrated phantom with known speed of sound, attenuation, and backscatter properties.

Methods have been proposed to estimate microstructural parameters of tissues based on tissue models, such as effective scatter diameter (ESD) and effective acoustic concentration (EAC) [88,93,94]. Typically, these methods involve solving an inverse equation problem, which involves fitting the measured parameters of BSC and AC into a pre-defined model with the scattering properties of the tissue of interest. Statistical models of backscattered echoes can provide an indirect estimation of the size and spatial distribution of the scatterers comprising tissue microstructure.

Studies have utilized raw RF data to research both healthy and diseased muscles. Ophir et al. measured the AC of the quadriceps muscles of healthy volunteers, demonstrating significantly different AC values among different individuals. They noted the range of AC values in normal individuals at 4.3 MHz to be 4.471 ± 0.44 dB/cm [95]. Zaidman et al. measured the effects of muscle contraction and probe orientation on normal muscle backscatter levels and compared backscatter levels between healthy and hereditary myopathy groups. They found backscatter levels to vary with probe orientation, age, muscle contraction, and pathology. Increased reliability was noted with a longitudinal probe orientation. Backscatter levels were found to be higher in subjects older than 40, myopathic patients, and muscle flexion [96]. In general, a common problem is the poor reproducibility of results from different ultrasound systems. Zaidman et al. developed a measurement called the calibrated muscle backscatter (cMB) which was found to have superior reliability (i.e., intraclass correlation coefficient) to gray-scale measurements when compared across different ultrasound systems [97]. In essence, calculation of the cMB is a two-step process, first requiring a backscatter vs. gray-scale level calibration curve to be created for each ultrasound system (correcting for between-system variations in the conversion between levels), and second, requiring reference to a commercial phantom during muscle scanning (correcting for variations in gain) [97].

It is informative to explore the relationships between QUS techniques and other methods used in the analysis of muscular diseases. Roy et al. investigated the relationships between electrical impedance myography (EIM) and both gray-scale level analysis (GLA) and quantitative backscatter analysis (QBA) in children with DMD. QBA provides direct analysis of the amplitudes of the reflected ultrasound echoes measured in decibels. A moderate correlation was found between QBA and EIM parameters when comparing the average of the muscles studied and when studying the biceps brachii and deltoid specifically, suggesting that QBA and EIM provide related data but are sensitive to different pathological features [98]. QBA and gray-scale levels measured from superficial regions of muscle were shown to similarly quantify muscle in DMD patients, although gray-scale levels measured from whole regions of muscle did not show significant increase in DMD patients [99]. Zaidman et al. assessed the changes in quantitative muscle ultrasound data in DMD patients and healthy individuals using both gray-scale level analysis and QBA. Both methods were found to perform similarly and more sensitively than functional assessments such as 6-min walk and supine-to-stand tests. Neither method showed differences in the subset of patients who initiated corticosteroids; thus, additional studies on QBA evaluation of therapeutic efficacy are warranted [100].

Santoso et al. investigated correlations between cervical smooth muscle force generation and ESD values. They found a significant positive correlation between muscle force generation and ESD immediately after oxytocin administration, suggesting ESD as a useful biomarker for studying cervical muscle contractility, an important factor for proper fetal development and delivery [101].

## 6. Envelope Statistics-Based Methods

The shape and attributes of the envelope of the backscattered signal contain information about the microstructure of the tissue [87]. Envelope statistics are computed by fitting a model distribution to the distribution of envelope amplitude of the RF data. For example, the Nakagami distribution models random distributions of a range of small to large number of scatterers in the resolution cells [102]. Other notable models include the Rayleigh, Rician, gamma, and K distributions. The homodyned K distribution is a more general model, in that the other distributions are special cases of it, but it is more complex to apply [103].

Tsui et al. used the Nakagami factor estimated from the statistical distribution of the backscattered ultrasound signals from vocal folds as a means for providing information about biomechanical properties. The textures of the Nakagami images of the lamina propria and vocal cord muscle of each vocal fold were compared. There were differences between the Nakagami parameters of the lamina propria and the vocal cord muscles, and different shading features were seen in the parametric maps (images) of the Nakagami factor. The Nakagami parameter may depend on the concentration of collagen and elastic fibers [104]. Weng et al. used the Nakagami distribution techniques to evaluate DMD. They studied healthy individuals and patients with varying stages of DMD. They found the Nakagami parameter was positively correlated with functional severity in DMD patients. They found the biggest difference between normal and stage 3 DMD patients and found the Nakagami parameter for the gastrocnemius muscle to correlate negatively with walking distance tests in ambulatory patients. The Nakagami parameter may be useful in monitoring disease progression in ambulatory patients [105].

Sikdar et al. investigated the use of a mixture of gamma distributions of backscattered US signal in analyzing muscles of healthy children and those with cerebral palsy (CP). Significant differences were quantified between the healthy and CP groups, with a more disorganized architecture and echogenicity in the CP muscles, which was likely due to fibrous infiltration, suggesting that these methods may be used to objectively differentiate muscle architecture and tissue properties [106].

Goryachev et al. investigated multiple envelope statistical QUS metrics to differentiate muscles in healthy, dystrophic, and obese mice. Among the different methods used, the homodyned K distribution provided the best accuracy. They conclude that dystrophic and obese mice have muscles with distinct acoustic properties that can be accurately classified using these methods [107].

The entropy of the amplitude of the backscattered echo can be utilized as a model-free technique for analyzing the statistics of the backscattered echo [108]. In general, model-free techniques do not offer a direct estimation of the microstructure of the tissue, but may remain useful as there are relationships between coefficients and microstructural parameters. Yan et al. used small-window entropy as a non-model-based approach to characterize muscle tissue in DMD. They found that entropy imaging can visualize changes in the information uncertainty of backscattered signals, with entropy value differences found among the different DMD disease stages and control groups. Comparisons to prior studies showed that the small-window entropy technique exhibits higher diagnostic performance than conventional methods [109].

## 7. Quantitative Ultrasound Limitations in Skeletal Muscle Evaluation

There are shared limitations between both qualitative and quantitative ultrasound which may include acoustic shadowing, differing insonation angles, reverberation, and clutter artifacts. In addition, there are limitations due to variations in system settings and parameters, including RF frequency, sampling rate, post-processing filters, and gains, which may lead to biased results. Some quantitative ultrasound methods are difficult to compare across different machines, which makes large multi-center studies difficult and highlights the utility of muscle-specific systems [110,111]. In more homogeneous tissues such as the liver, BSC and AC were found to be highly repeatable and reproducible across scanners and operators [112], but such studies in muscle are few. Additional considerations may include the availability and cost of these techniques. Multiple studies emphasize the importance of scanning protocols and note that despite the relative objectivity of QUS, there is still a notable degree of operator dependence. 

Each of the techniques discussed in this review has limitations that may be shared or unique to a specific QUS technique. B-mode echogenicity measurements achieved after post-processing may be limited due to their high dependence on scanner type, settings, and manual boundary identification [61,67]. The transducer frequency and device gain may affect the hyperechoic fractions [35]. Studies have also found significant differences in ultrasound B-mode images due to overlying abdominal wall thickness and composition, especially when using a high frequency transducer [113]. Automatic thresholding methods for B-mode echogenicity measurements may also be affected by differences in ultrasound equipment; therefore, there is a need for studies with various US machines [33]. B-mode echo intensity measurements across two different ultrasound machines have been completed successfully on healthy individuals using a dedicated phantom and a conversion equation, which allowed normal values obtained with one device to be comparable to another device. However, further studies are warranted as the robustness of this method is in question since it requires specific phantoms and relative avoidance of additional post-processing [114]. In addition, echogenicity measurements alone may not be enough to differentiate the precise biological or physiological process that is occurring. Consideration of the chronicity of change is important [115]. Lastly, additional techniques such as texture analysis may be useful in some settings, such as differentiating between fatty infiltration and fibrosis, both of which increase muscle echogenicity [27].

Elastography techniques may be limited by a lack of well-accessible muscles and poor time-efficiency [85]. Muscle stiffness is also affected by the degree of muscle contractility and joint position. Due to these factors, elastography may require complementary tools such as electromyography. The transducer pressure and the equal application of pressure along the transducer length during stress and shear wave elastography can affect SWV values. Strain elastography specifically suffers from the eggshell effect where harder tissues at the boundary of tissues can be difficult to deform, thus limiting the analyzation of internal strain [116]. Some studies also suggest that elastography is dependent on the studied muscle’s pennation angle [117]. When compared with traditional material testing techniques, SWE correlates well when the transducer is parallel to the underlying muscle fibers, highlighting that standardization of elastography protocols is important [83,118,119]. SWE has limited depth of penetration, and SWV values also vary by depth and ROI proximity to stiffer materials such as tendon tissues or calcium, so further development of protocols that are consistently followed is important [77]. Elastography models often assume a linear and purely elastic material; however, biological tissues are typically nonlinear, viscoelastic, and anisotropic [120]. It is also worth noting that since shear waves are slow moving, motion of the patient or transducer reduces reliability. There are also limitations to drawing proper elastography ROIs of the muscle belly while trying to reduce noise from adjacent bone artifact [121].

Backscatter RF signal characterization with the reference phantom technique depends on the analyzation of tissues relative to a calibration phantom, which has a known BSC profile in the range of targeting frequencies. The phantoms are required to be well-calibrated, stable over time, and readily accessible during the scan session. RF signal characterization also requires more advanced data processing, often offline, when compared with other QUS methods. It is also worth noting that most analytical methods to evaluate attenuation assume the medium is homogenous, whereas in vivo samples are often heterogeneous. As previously noted, the backscattered-signal model-based methods require solving an inverse equation by fitting the measured parameters of BSC and AC into a pre-defined model with the scattering properties of the tissue of interest. Similar to other QUS methods, the RF signal characterization can be affected by transducer orientation and applied compression pressure [96].

Envelope statistics-based methods have varying levels of computational complexity depending on the model utilized. However, with advancements in ultrasound system processing and memory, nearly all QUS methods should be feasible for implementation on clinical scanners including point-of-care devices [87].

## 8. Conclusions

Muscle evaluation with QUS include B-mode quantitative techniques, ultrasound elastography, radiofrequency spectral characterization, and envelope statistics-based methods. The literature provides myriad potential uses of QUS in evaluating healthy muscles, diagnosing pathological conditions, and directing and monitoring treatments. Although the goal of QUS is to provide relatively objective quantitative biomarkers for evaluating muscles when compared with traditional qualitative methods, there are still issues to address including operator and system repeatability and reproducibility. Therefore, additional research is vital, especially research with a focus on standardization of scanning methods, data analysis, and the elucidation of quantitative value averages and ranges of various muscles in healthy and diseased states.

## Data Availability

No new data was created during this study.
